# Barriers and Facilitators of Indoor No-Smoking Policy Implementation Among Older Chinese American Women

**DOI:** 10.1093/geroni/igaf122.2389

**Published:** 2025-12-31

**Authors:** Chien-Ching Li, Tingfei Hu, Samantha Wu, Sharon Mei, Alicia K Matthews

**Affiliations:** Rush University, Chicago, Illinois, United States; Rush University, Chicago, Illinois, United States; University of Illinois Chicago, Chicago, Illinois, United States; University of Illinois Chicago, Chicago, Illinois, United States; Columbia University, New York, New York, United States

## Abstract

**Objective:**

Asian American female non-smokers face nearly twice the risk of lung cancer compared to their White counterparts. Among Chinese Americans, smoking is predominantly a male behavior, influenced by cultural norms, with approximately 25% of Chinese American men being current smokers. Given that over 88% of them are married, Chinese American women face heightened exposure to secondhand smoke (SHS), a known risk factor for lung cancer. Family and cultural dynamics may shape SHS exposure patterns, influencing the adoption of indoor no-smoking policies. This study examines the barriers and facilitators to implementing such policies among Chinese American women using the Health Belief Model.

**Methods:**

Our study recruited 86 Chinese American women aged 50 and older who had ever lived with a smoker. Participants completed a survey assessing indoor smoking policies at home, with selected questions developed based on the Health Belief Model. The survey measured perceived susceptibility to secondhand smoke (SHS), perceived severity of SHS, perceived benefits of a smoke-free environment, self-efficacy in implementing no-smoking policies, and cues to action.

**Results:**

Binary logistic regression results showed that Chinese women who expressed higher levels of self-efficacy were 1.8 times more likely to implement no-smoking policies at home after controlled for covariates.

**Conclusion:**

Higher self-efficacy significantly increased the likelihood of implementing indoor no-smoking policies among Chinese American women. Culturally tailored interventions that enhance self-efficacy may be key to reducing secondhand smoke exposure and lung cancer risk in this population.

